# Decoupling dedifferentiation and G_2_/M arrest in kidney fibrosis

**DOI:** 10.1172/JCI163846

**Published:** 2022-12-01

**Authors:** Benjamin D. Humphreys

**Affiliations:** 1 Division of Nephrology, Department of Medicine and; 2 Department of Developmental Biology, Washington University in St. Louis, St. Louis, Missouri, USA.

## Abstract

Understanding the cellular mechanisms underlying chronic kidney disease (CKD) progression is required to develop effective therapeutic approaches. In this issue of the *JCI*, Taguchi, Elias, et al. explore the relationship between cyclin G1 (CG1), an atypical cyclin that induces G_2_/M proximal tubule cell cycle arrest, and epithelial dedifferentiation during fibrogenesis. While CG1-knockout mice were protected from fibrosis and had reduced G_2_/M arrest, protection was unexpectedly independent of induction of G_2_/M arrest. Rather, CG1 drove fibrosis by regulating maladaptive dedifferentiation in a CDK5-dependent mechanism. These findings highlight the importance of maladaptive epithelial dedifferentiation in kidney fibrogenesis and identify CG1/CDK5 signaling as a therapeutic target in CKD progression.

## The proximal tubule in the AKI-to-CKD transition

Chronic kidney disease (CKD) affects approximately 800 million people worldwide and treatment options are limited (1). CKD is characterized by tubular atrophy, inflammation, interstitial fibrosis, and progressive loss of kidney function. One common cause of CKD is acute kidney injury (AKI) in a process termed the AKI-to-CKD transition. In the past, it had been thought that the kidney completely recovered after an episode of AKI, but over the last 15 years it has become clear that complete recovery is likely the exception — some degree of permanent damage and fibrosis exists after every episode of AKI, even if subclinical.

The molecular mechanisms regulating the AKI-to-CKD transition remain incompletely understood. In successful repair, injury induces cellular dedifferentiation, characterized by the loss of brush border and terminal differentiation markers (Sox9 and vimentin [VIM]) and the acquisition of a transient mesenchymal phenotype. This process is followed by cellular proliferation to replace neighboring epithelia lost through cell death, followed by redifferentiation and restoration of tubular function. Many studies have implicated aberrant proximal tubule injury responses as a central driver of the AKI-to-CKD transition (2, 3). Some results indicate that a subset of the injured proximal tubules become arrested in the G_2_/M phase of the cell cycle, leading to adoption of a senescence-associated secretory phenotype (SASP) (4, 5). This proinflammatory cell state promotes local inflammation and fibrosis that lead to CKD. Other work has identified a subset of dedifferentiated epithelia that fail to redifferentiate after injury, instead adopting a proinflammatory and profibrotic state variously termed “maladaptive” or “failed repair” (6–9). Importantly, it is not yet clear whether G_2_/M-arrested cells and failed-repair epithelia are one and the same, although a parsimonious interpretation of the literature would indicate that they should be. Casting doubt on this hypothesis, recent single-cell RNA sequencing studies have suggested that failed-repair proximal tubule cells are not in a G_2_/M-arrested state, suggesting distinct proximal tubule cell states exist (6, 7).

## Proximal tubule dedifferentiation and the CG1/CDK5 pathway

In this issue of the *JCI*, Taguchi, Elias, et al. (10) shed light on the relationship between proximal tubule dedifferentiation, G_2_/M arrest, and the AKI-to-CKD transition. The authors had previously identified cyclin G1 (CG1), an atypical cyclin induced by p53 and known to regulate G_2_/M arrest in other contexts, as a key player in the AKI-to-CKD transition (5). In particular, they concluded that CG1 is specifically induced in the maladaptive proximal tubule and that it is sufficient to induce both cellular dedifferentiation and G_2_/M arrest that lead to CKD. In their current work, the authors subjected global CG1–knockout mice, which lack a kidney phenotype in health, to various models of the AKI-to-CKD transition. Knockout mice were clearly protected from the development of CKD after AKI, consistent with the proposed role of CG1 in promoting G_2_/M arrest and fibrosis (10).

The authors reasoned that if CG1 promotes CKD by inducing G_2_/M arrest, then inducing G_2_/M arrest should reverse the protective effect of CG1 knockout. Unexpectedly, knockout mice exposed to paclitaxel were still protected from CKD, despite the strong induction of G_2_/M arrest. These findings suggest that CG1 regulates proximal tubule fibrosis but independent of G_2_/M arrest. Follow-up studies showed that CG1-knockout kidneys were characterized by less dedifferentiation and proliferation of proximal tubules in the chronic injury phase, suggesting that rather than drive fibrosis by inducing G_2_/M arrest, CG1 may be driving fibrosis by inducing proximal tubule dedifferentiation (10).

**Figure 1:**
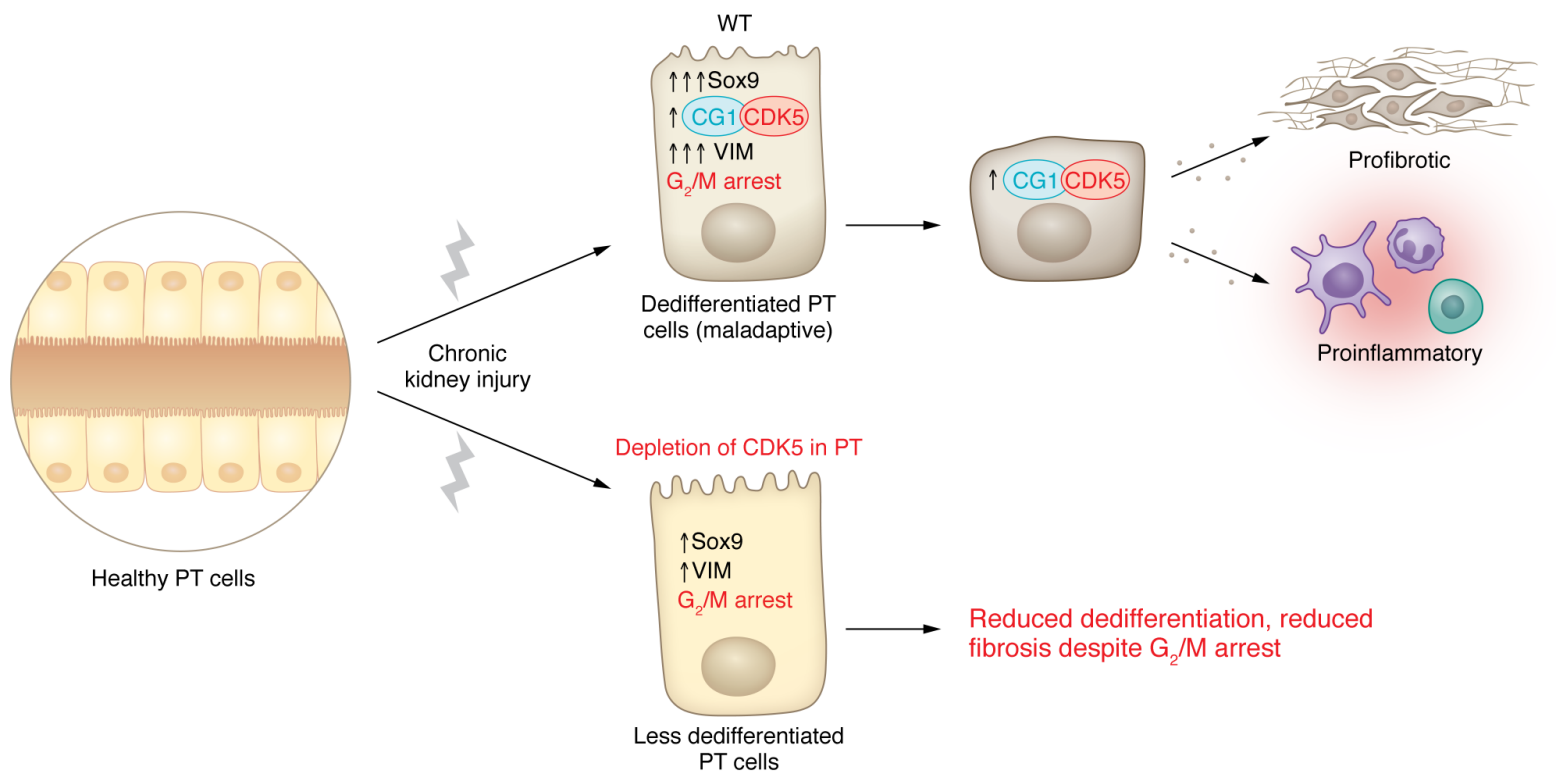
The CG1/CDK5 pathway is involved in regulating proximal tubule dedifferentiation and fibrosis in CKD. Chronic kidney injury induces cells in the proximal tubule (PT) to undergo maladaptive dedifferentiation with a G_2_/M cell cycle–arrested phenotype. These cells also show high Sox9 and VIM expression and activation of the CG1/CDK5 pathway. Ensuing proinflammatory and profibrotic signaling results in fibrosis and decreased kidney function. Compared with WT CKD models, PTs lacking CDK5 have reduced dedifferentiation, characterized by reduced Sox9 and VIM expression. However, cells without CKD5 remain arrested in the G_2_/M phase of the cell cycle, similarly to WT cells. Notably, the resultant phenotype displays reduced fibrosis with CKD despite induction of G_2_/M cell cycle arrest (10).

CG1 activates cyclin-dependent kinase 5 (CDK5) through phosphorylation of tyrosine 15, and phosphorylated CDK5 was detectable in maladaptive proximal tubules of wild-type but not CG1-knockout kidneys. In vitro studies showed that CDK5 expression was sufficient to drive proximal tubule dedifferentiation, suggesting that CDK5 activation mediates CG1-dependent dedifferentiation. Since CG1-knockout mice were protected from the AKI-to-CKD transition, Taguchi, Elias, and colleagues next asked whether CDK5 is also a therapeutic target. They showed convincingly that indeed either pharmacologic inhibition of CDK5 with two different drugs or tubule-specific knockout of *Cdk5* protected against development of CKD after AKI. Importantly, *Cdk5* knockout reduced proximal tubule dedifferentiation, but did not reduce induction of G_2_/M arrest, further emphasizing a decoupling between dedifferentiation and G_2_/M arrest in the AKI-to-CKD transition (Figure 1) (10).

## Implications and unanswered questions

There are several implications of this work. Perhaps most importantly, these studies shift focus away from G_2_/M arrest as a central, required cell state for the development of fibrosis and CKD, as had been concluded previously. Deletion of CG1 inhibited both G_2_/M arrest and fibrosis but subsequent induction of G_2_/M arrest did not reverse this protective phenotype, providing strong evidence that, in this context at least, proximal tubule G_2_/M arrest was not sufficient to drive fibrogenesis (10). Instead, proximal tubule dedifferentiation in CKD appears to be the critical profibrogenic cell state, one that is regulated by the CG1/CDK5 axis. It is likely that G_2_/M arrest still plays roles in CKD, perhaps acting in addition to other profibrotic pathways rather than as a necessary and sufficient state.

Another intriguing implication of Taguchi, Elias, et al. (10) that clearly needs further investigation is the suggestion that proximal tubule dedifferentiation after AKI may be fundamentally different from dedifferentiation after CKD. While both acute and chronic injuries lead to the loss of brush border and differentiation markers, which superficially resemble equivalent dedifferentiation events, neither CG1 nor CDK5 was required for successful proximal tubule repair (10). By contrast, this signaling pathway plays a critical role in driving epithelial dedifferentiation and fibrosis in CKD. Similarly, what roles, if any, do CG1 and CDK5 play in the AKI-to-CKD transition? In mild or moderate AKI, the majority of proximal tubule cells successfully proliferate and redifferentiate after injury (11), processes that this work (10) shows do not require CG1 or CDK5. But left unresolved is whether the fraction of proximal tubule cells (~5%–10%) that take on a “failed repair” or “maladaptive” cell state after AKI do so as a consequence of activation of the CG1/CDK5 pathway. It stands to reason that they may well be related, since single-nucleus RNA sequencing of AKI-to-CKD models demonstrates that this minority cell population is not arrested at G_2_/M. These questions also await further experimental investigation.

That profibrotic cellular dedifferentiation in CKD can be targeted therapeutically by inhibition of CDK5 not only validates the kinase as a therapeutic target in fibrosis, but also suggests that other downstream pathways could represent additional antifibrotic targets as well. The signaling pathways either upstream of CG1 or downstream of CDK5 in proximal tubules remain undefined. Mitochondrial dynamics and dysfunction are increasingly recognized to play critical roles in driving both recovery from AKI as well as the progression of CKD (12). Mitochondrial dysfunction can lead to leakage of mitochondrial DNA into the cytosol, where it activates the cytosolic cGAS-stimulator of interferon genes (STING) DNA sensing pathway that then drives proinflammatory cytokine expression and renal fibrosis (13). In the brain, CDK5 has established roles in promoting mitochondrial fission and dysfunction and in some neuronal cell types this pathology leads to cell death (14). Given this context, it is intriguing to speculate that the CG1/CDK5 axis may link profibrotic epithelial dedifferentiation to mitochondrial dysfunction, inflammation, and fibrosis. For example, CDK5 phosphorylates the GTPase dynamin-related protein 1 (Drp1), and this phosphorylation at S616 increases Drp1 translocation to the mitochondria, accelerating fission (15, 16). Proximal tubule–specific deletion of Drp1 promotes recovery after AKI (17), suggesting that a CDK5-dependent phosphorylation of Drp1 in CKD may cause mitochondrial fission, mitochondrial dysfunction, and potentially renal inflammation and fibrosis through the STING pathway. All of these hypotheses require testing.

In summary, Taguchi, Elias, and colleagues uncouple G_2_/M arrest from dedifferentiation and progression of fibrosis. They implicate a CG1/CDK5 signaling axis in regulating proximal tubule dedifferentiation and fibrosis and validate these proteins as therapeutic targets in CKD (10).

## Version 1 12/01/2022

Electronic publication
